# Sero-epidemiological investigation on *Toxoplasma gondii* infection in Apennine wolf (*Canis lupus italicus*) and wild boar (*Sus scrofa*) in Italy

**DOI:** 10.1186/s12917-024-03922-2

**Published:** 2024-02-22

**Authors:** Filippo Maria Dini, Carmela Musto, Vincenzo Maria De Nigris, Enrica Bellinello, Maria Sampieri, Giuseppe Merialdi, Lorella Barca, Mauro Delogu, Roberta Galuppi

**Affiliations:** 1 Department of Veterinary Medical Sciences, University of Bologna, Ozzano Emilia, Bologna, 40064 Italy; 2 AUSL Bologna dipartimento di Sanità Pubblica Veterinaria- UO Veterinaria B, Via del Seminario, 1 San Lazzaro di Savena, Bologna, Italy; 3 AUSL Modena, dipartimento di Sanità Pubblica Veterinaria, via Suore di San Giuseppe Benedetto Cottolengo, 5 41026 Pavullo nel Frignano, Modena, Italy; 4 Istituto Zooprofilattico Sperimentale della Lombardia e dell’Emilia-Romagna Bruno Ubertini, Brescia, Italy; 5 Istituto Zooprofilattico Sperimentale del Mezzogiorno, Portici, Napoli, Italy

**Keywords:** Toxoplasmosis, Wildlife, Epidemiology, Serology, IFAT

## Abstract

**Background:**

The wild boar (*Sus scrofa*) and the Apennine wolf (*Canis lupus italicus*) are two wild species that have both increased their presence in the Italian territory, albeit in varying numbers. They can be occasionally found in peri-urban areas as well. Both of these species can serve as intermediate hosts for *Toxoplasma gondii*, as they can become infected either through the consumption of oocysts found in water, soil, or on vegetables, or through the ingestion of meat containing bradyzoites. Consequently, these animals can be regarded as key indicators of *Toxoplasma* presence in the wild or peri-urban environment. In our study, we examined a total of 174 wild boar meat juice and 128 wolf sera from Italy for the detection of *T. gondii* IgG using the indirect fluorescent antibody test (IFAT).

**Results:**

The results showed that 40 (22.6%) of the wild boar meat juice and 34 (26.6%) of the wolf serum samples tested positive. Interestingly, there were no significant differences in seropositivity with respect to gender, age group, or the region of origin in both species.

**Conclusions:**

Overall the results indicate a moderate exposure in both the species under investigation, highlighting the spread of *T. gondii* in sylvatic and periurban environments. The prevalence of *T. gondii* in wild boar is consistent with findings from other studies conducted in Europe. Our study, with a considerably larger sample size compared to the available research in European context, provides valuable data on the seroprevalence of *T. gondii* in wolves.

## Background

*Toxoplasma gondii* is a globally distributed apicomplexan protozoan. Its widespread epidemiological success can be attributed to its ability to infect both definitive and intermediate hosts through various modes [1].

Definitive hosts, primarily members of the Felidae family, facilitate the parasite’s sexual reproduction in their intestinal tract, potentially leading to the excretion of millions of oocysts into the environment. In our regions wild and domestic cats play a crucial role in perpetuating this parasite [2]. In Italy, the native European wildcat (*Felis silvestris silvestris*) maintains a relatively small population size in the wild, despite beingclassified as Least Concern in the IUCN Red List of Threatened Species [3]. However, free-roaming domestic cats are prevalent in rural and peri-urban regions [4].

All warm-blooded vertebrates, including humans, can serve as intermediate hosts in which cysts housing long-lasting bradyzoites develop. These hosts become infected by ingesting sporulated oocysts, although the parasite may persist through predation among them, even in the absence of a definitive host [1].

Omnivorous wild boars (*Sus scrofa*) are susceptible to infection through two plausible routes: ingestion of highly resistant oocysts present in water and vegetation, and consumption of remains of infected intermediate hosts [5]. Additionally, wild boars represent a potential risk to human health through the consumption of raw or undercooked game meat [6]. The wolf (*Canis lupus*) can also act as an intermediate host of *T gondii*. Despite the wolf’s primarily carnivorous diet, which includes predation on live animals, including wild boar, it has been established that they also frequently consume fruits (Rosaceae), other plant matter, and insects [7]. Consequently, both modess of infection are viable in these animals, positioning them at the apex of receptive intermediate hosts range.

The IUCN Red List of Threatened Species has classified the European assessment of *Canis lupus* as “Least Concern” [8]. In Italy, a subspecies of the grey wolf known as the Apennine wolf (*Canis lupus italicus*) has seen a population expansion throughout the Italian peninsula in recent years [9], with the exception of the islands. Over the past few decades, both the number and distribution of wolf populations in Italy have increased. Wolves have been progressively reclaiming their historic habitats, moving from the Apennines to the western areas of the Italian Alps [10, 11]. In the past decade, they have also expanded into the eastern Alps [12]. While wolves tend to prefer locations at a considerable distance from human settlements, they have been observed in close proximity to urban areas in densely populated regions [13].”

Despite being among the most heavily hunted ungulate species, wild boars have undergone a population expansion throughout Europe. In Italy, the density of wild boars has been estimated to range from 0.01 to 0.05 animals per square kilometer, increasing to as high as 2.32 to 10.5 animals per square kilometre across the entire Italian peninsula [14]. The simultaneous expansion of human-inhabited areas and the wild boar populations has facilitated the intrusion of this species into various European urban areas, including Rome [15].

In the present study, we conducted a serological survey on wolves and wild boars from different regions of Italy. The objective was to gather data on their exposure to *T. gondii* infection, serving as indicators of *Toxoplasma* presence within the wild or peri-urban environment.

## Results

The wild boars displayed nearly equal representation across sex and age groups, with a notable portion originating from the Tuscany region (as reported in Table 1). Among the 177 meat juice samples, 40 (22.6%) tested positive for *Toxoplasma* IgG at IFAT. No statistically significant differences of seropositivity were observed in relation to sex, age groups and region of origin and between wolves and wild boar.

**Table 1:** Descriptive statistics and serological tests result in wild boar examined

	Category	n. wild boar tested	Relative distribution %	IFAT positive	Seroprevalence %	95%CI	
Total		177		40	22.6	[16.44–28.76]	
Gender	Male	83	53.2	14	16.9	[8.84–24.96]	
	Female	73	46.8	21	28.8	[18.41–39.19]	
Age groups	Young	92	52.3	25	27.2	[18.11–36.29]	
	Elderly	84	47.7	14	16.7	[8.72–24.68]	
Region of origin	Tuscany	76	42.9	15	19.7	[10.76–28.64]	
	Emilia Romagna	51	28.8	9	17.6	[7.15–28.05]	
	Abruzzo	48	27.2	16	33.3	[20–46.63]	
	Molise	2	1.1	0	0	[]	

*Note* In 21 cases, the sex of the subjects could not be ascertained, and in one case, the age was unknown, due to incomplete filling of the animal’s identification form

The region of origin of wolves and their cause of death are summarized in Table 2). Thirty-four (26.6%) out of 128 serum analysed, were positive at IFAT, with antibody titres ranging from 1:20 to 1:160. It is noteworthy that no statistically significant differences were observed to seropositivity in relation to sex, age group, geographic origin or cause of death.

**Table 2:** Descriptive statistics and serological test results in wolf examined

	Category	n. wolf tested	Relative distribution %	IFAT positive	Seroprevalence %	95%CI	
Total		128		34	26.6	[18.95–34.25]	
Sex	Male	79	62.2	26	32.9	[22.54–43.26]	
	Female	48	37.8	8	16.7	[6.15–27.25]	
Age class	1: <12 months	42	33.1	10	23.8	[10.92–36.68]	
	2: 1–2 years	31	24.4	5	16.1	[3.16–29.04]	
	3: > 2 years	54	42.18	19	35.19	[24.87–45.51]	
Region of origin	Tuscany	63	49.2	21	33.3	[21.66–44.94]	
	Emilia-Romagna	45	35.2	11	24.4	[11.85–36.95]	
	Calabria	15	11.7	2	13.3	[0–31.1]	
	Umbria	3	2.3	0	0	[]	
	Veneto	2	1.6	0	0	[]	
Cause of death	Car crash	75	60.5	16	21.3	[12.3–30.57]	
	Other cause	49	39.5	16	32.6	[19.4–45.8]	

*Note* In one subject it was not possible to know the gender and in one the age due to the poor condition of the carcasses. In four subjects (two positive and two negative) the cause of death was undetermined

**Fig. 1:**
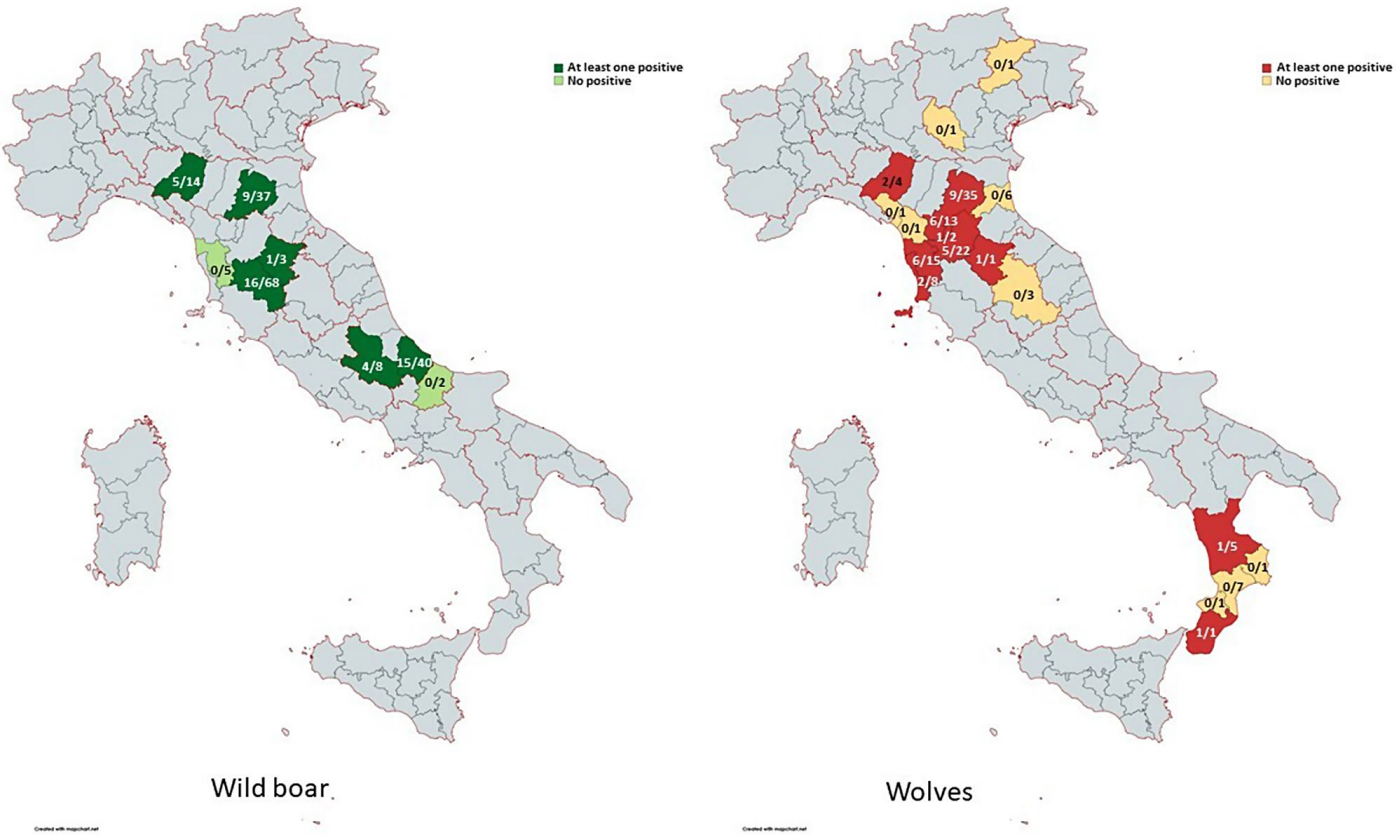
Distribution of wild boar and wolves examined; number positive/number examined

In Fig. 1 the distribution of wolves and wild boar examined are illustrated, with the number of seropositive/number of examined samples in the different Italian provinces.

In the geographical areas where there was an overlap in the sampling of wild boars and wolves (Emilia-Romagna and Tuscany regions), the seroprevalences were 18.9% and 29.6%, respectively, even though the differences were not significant.

## Discussion

In this study, we evaluated the seroprevalence of *T. gondii* in two species, wild boar and wolves. Despite their role as intermediate hosts, these species could play a significant role in mantaining the effective continuity of the parasite’s life cycle in the wild. Both these animals can become infected through the ingestion of robust, environmentally enduring oocysts, as well as via the consumption of prey or carrion. Consequently, they serve as valuable indicators to detect the presence of *T. gondii* contamination within specific ecological contexts [16, 17].

During this study, we utilized two different matrices: serum samples from wolves and meat juice from wild boar. The choice of these two matrices was driven by practical considerations. In the case of wolves, which were found deceased, we were able to conduct a comprehensive necropsy, including the collection of clotted blood from the heart cavity and subsequent extraction of serum. On the other hand, for wild boars, a different approach was necessary. These animals were hunted and eviscerated before slaughtering, making it impossible to collect blood directly.Therefore, we chose meat juice as a more appropriate and easily accessible matrix in this situation.

This matrix has been used in previous studies for the detection of antibodies against *T. gondii* [18] as well as other zoonotic pathogens such as *Trichinella* sp. [19, 20], *Salmonella* sp., and Hepatitis E virus [21]. The use of meat juice as a matrix is particularly advantageous, as it can be easily obtained from wildlife carcasses, often found deceased, thereby providing valuable serological data that would otherwise be challenging to collect. However, it’s important to note that meat juice has been perceived as a matrix with lower sensitivity in comparison to serum, primarily due to the lower antibody concentration it contains [22]. While serological data derived from either sera or meat juice samples offer insights into an animal’s exposure to the parasite, they do not provide information concerning the presence of tissue cysts within organs, which directly relates to the risk for consumers [23, 24].

In the present study, an overall seroprevalence rate of 22.6% was observed in wild boars (ranging from 0 to 33.3% accross the different regions), and no statistically significant differences were observed among the variables considered, including age, in line with the findings of some authors [25–27]. Recent meta-analyses have shown that the global pooled seroprevalence of *T. gondii* in wild boars from 1995 to 2017 was 23%, which aligns closely with our findings [17]. However, various seroprevalence rates have been documented on wild boars in different geographical settings. For instance, in Europe, seroprevalence values ranging from 8 to 38% have been reported [17, 28–30]. Specifically, surveys conducted in central and southern regions of Italy, reported values ranging from 12.2% [31] to 14% [21, 32], while recent surveys in Northern Italy have identified seroprevalences spanning from 15.5% [33] to as high as 53.1% [27]. These seroprevalence differences could be related to specific local epidemiological conditions, such as variations in environmental factors, wildlife populations, or human activities, highlighting the importance of considering local risk factors in understanding the epidemiology of Toxoplasmosis.

In wolves, a seroprevalence rate of 26.6% was observed in this study. When comparing seroprevalences between wolves and wild boars, despite wolves occupying higher trophic levels and exhibiting a higher prevalence of *T. gondii*, no statistically significant differences were observed between these two populations. This finding aligns with the results of Dakraub et al. [4]. Reliable *T. gondii* seroprevalence data for wolves in European countries, including Italy, are notably scarce. Recent reports from Italy have indeed documented seropositivity in wolves, albeit with relatively small sample sizes: Dini et al. [33] identified one positive wolf out of 5 samples, while Dakraub et al. [4] reported 4 positives out of 14. In other European countries, such as Spain, a seroprevalence rate of 46.9% was observed (*n* = 32 wolves sampled) [34]. Due to the considerable higher sample size, the present study offers a comprehensive assessment of *T. gondii* seroprevalence in wolves, thereby contributing valuable data on a European scale.

In addition to its epidemiological significance, seropositivity in wolves has been associated with ecological implications, particularly in the United States. Recent research [35] demonstrated that the overlap of wolf territories with regions characterized by a high cougar population density serves as a significant predictor of *T. gondii* infection in wolves. Furthermore, wolves that tested positive through serological analysis were found to be more inclined to make high-risk decisions, such as dispersing and assuming leadership roles within packs [35]. These decisions have a pivotal impact on individual fitness and the broader dynamics of wolf. In the current study, we did not observe a positive correlation between seropositivity and the cause of death being a car crash. Instead, even when considering seropositivity as a factor contributing to increased wolf dispersion, it does not appear to be linked to car collision as cause of death in our sample set.

## Conclusion

This study provides an update on the spread of *T. gondii* in sylvatic and peri urban settings, highlining a moderate exposure in both the species under investigation. Additional research endeavours should be undertaken to explore the correlation between *T. gondii* seropositivity in wolves and factors like dispersal rates, causes of death, and spatial overlap with other species, including humans. These studies will be able to contribute to a more comprehensive understanding of the significance of *T. gondii* seroprevalence, including its ecological implications.

## Methods

Approximately 25 g of diaphragm tissue from wild boars were systematically collected at a specialized game meat processing facility located in the Bologna province (Emilia-Romagna region). This facility routinely receives eviscerated carcasses of hunted wild boars from various regions of Italy, encompassing Emilia-Romagna, Tuscany, and Abruzzo. Sex, and age class were determined, the latter assessed by the evaluation of the dental table. The diagnostic matrix employed in this study was the meat juice, as carcasses have already been bled and eviscerated. To extract the meat juice from the diaphragm tissue, the samples were placed in hermetically sealed plastic container, and frozen at -20 °C. Following this step, the meat samples were thawed over-night, at a controlled temperature of 4 °C. The resulting meat juice was then transferred into sterile tubes, preserved at -20 °C until use [36].

The examined wolves came mainly from Toscana and Emilia-Romagna region (Central Italy), in less extent they were collected from Calabria (south), Umbria (centre), and Veneto (north) regions. The wolves were found dead and delivered to authorized centers in order to proceed with the necropsy. Necropsy examinations on wolf carcasses were carried out at the Experimental Zooprophylactic Institute of Lombardy and Emilia-Romagna, the Wildlife and Exotic Service of the University of Bologna and at the Experimental Zooprophylactic Institute of Southern Italy. At the arrival of each carcass, a first form containing the following information was filled: subject’s identification data with the attribution of a unique ID code, the discovery location (reported as GPS coordinates), sex, weight (in kg) and nutritional status. The age of the animal was determined by assessing dental development and wear [37, 38], as well as considering body size and weight. Here, all individuals were aged using 3 categories as follows: class 1: ≤12 months; class 2: 1–2 years; class 3: > 2 years. The age determination of class 1 (based on months of life) was defined in relation to the reproductive cycle of the wolf [39]. Besides the biometrics information, phenotypic characteristics and anatomopathological activities were carried out to investigate the cause of death [40]. During necropsy the entire heart was collected, and the heart blood clot was extracted and centrifuged at 980 g for 20 min. The haemolytic serum was then collected in a 2 ml tube and stored at -20 °C until use.

A total of 177 meat juices of wild boars and 128 wolf sera were analysed for *T. gondii* IgG by indirect fluorescent antibody test (IFAT) following the manufacturer’s instructions (MegaFLUO TOXO-PLASMA g, MegaCor Diagnostik, Hoerbranz, Austria). As conjugated, anti-dog IgG antibody diluted in PBS at concentration of 1:64 (Anti-Dog IgG-FITC antibody, Sigma-Aldrich, Saint Louis, MO) and anti-pig. IgG antibody diluted in PBS at concentration of 1:32 (Anti-pig IgG-FITC antibody, Sigma-Aldrich, Saint Louis, MO) were used. Meat juice from wild boars with an antibody titre ≥ 1:4 were considered positive (due to the scarce concentration of antibody in this matrix) [22], while wolf serum samples with antibody titre ≥ 1:20 were considered positive (due to the haemolytic characteristics of the sera) [41].

Pearson’s χ2 test was used to correlate sex, age group, region of origin (and cause of death in wolf) with seroprevalence. Statistical significance was set at P ≤ 0.05. The Sample Size Calculator (https://www.surveysystem.com/sscalc.htm) was used to calculate 95% confidence intervals (CIs) for the observed prevalence values.

## Data Availability

The datasets used and/or analysed during the current study are available from the corresponding author on reasonable request.
